# Ecological insights into hematopoiesis regulation: unraveling the influence of gut microbiota

**DOI:** 10.1080/19490976.2024.2350784

**Published:** 2024-05-10

**Authors:** Kaiwen Zheng, Zhifeng Wei, Wei Li

**Affiliations:** a Cancer Center, the First Hospital of Jilin University, Changchun, China; b Department of Hematology, The First Hospital of Jilin University, Changchun, China

**Keywords:** Hematopoietic stem cell, gut microbiota, bone marrow niche

## Abstract

The gut microbiota constitutes a vast ecological system within the human body, forming a mutually interdependent entity with the host. In recent years, advancements in molecular biology technologies have provided a clearer understanding of the role of the gut microbiota. They not only influence the local immune status and metabolic functions of the host’s intestinal tract but also impact the functional transformation of hematopoietic stem cells (HSCs) through the gut-blood axis. In this review, we will discuss the role of the gut microbiota in influencing hematopoiesis. We analyze the interactions between HSCs and other cellular components, with a particular emphasis on the direct functional regulation of HSCs by the gut microbiota and their indirect influence through cellular components in the bone marrow microenvironment. Additionally, we propose potential control targets for signaling pathways triggered by the gut microbiota to regulate hematopoietic function, filling crucial knowledge gaps in the development of this research field.

## 1. Introduction

The hematopoietic system is one of the most essential and intricate systems in the body, serving as the origin of all blood cells. Hematopoiesis is the process by which these cells differentiate and mature into red blood cells, white blood cells, and platelets. These cells play a pivotal role in maintaining the body’s immune system and oxygen-carrying capacity. Hematopoiesis is a continuous process due to the constant loss of blood cells in response to various situations such as infections and blood loss. Additionally, blood cells have limited lifespans; for instance, mature red blood cells typically have a lifespan of approximately 120 days. The source of all blood cells is a rare population of cells known as hematopoietic stem cells (HSCs). HSCs are characterized by their unique ability to self-renew and differentiate into all blood cell types, placing them at the top of the hierarchy.

HSCs are primarily located within the bone marrow, a microenvironment that provides a protective niche for these stem cells, which is key to their regenerative potential. In essence, the HSCs niche consists of both cellular and non-cellular components that regulate the state of HSCs through various pathways, including inflammatory signals and metabolic state transitions.^1^ HSCs in the niche receive signals from endogenous and exogenous sources to adapt to different hematopoietic demands.

In the human body, the gut microbiota is an organ that has not been fully explored and is intricately connected with the body, forming a mutually dependent entity.^2^ The dynamic balance of gut microbiota is crucial for maintaining health and is influenced by various external factors, affecting the body’s immune, nutritional, and metabolic processes.^3^ Several studies have suggested that the gut microbiota plays a partial role in regulating hematopoiesis, including compelling evidence for its role in regulating graft-versus-host disease (GVHD) after hematopoietic stem cell transplantation (HSCT). The impact of the gut microbiota on the hematopoietic system is gradually being recognized, and targeting microbiota dysbiosis should be a focus of future research. The application of gut microbiota in the regulation of hematopoietic function also requires in-depth investigation. In this article, we will explore the advancements in this emerging field of research that focuses on the interplay between the gut microbiota and the hematopoietic system. We will delve into how microbiota imbalance affects the functionality of HSCs and how these discoveries may open new avenues for future medical research and treatment. This offers fresh prospects for the development of disease treatments and stem cell therapies.

## 2. Hematopoietic stem cell and BM niche

### 2.1. Development of hematopoietic stem cells

**Figure 1.**
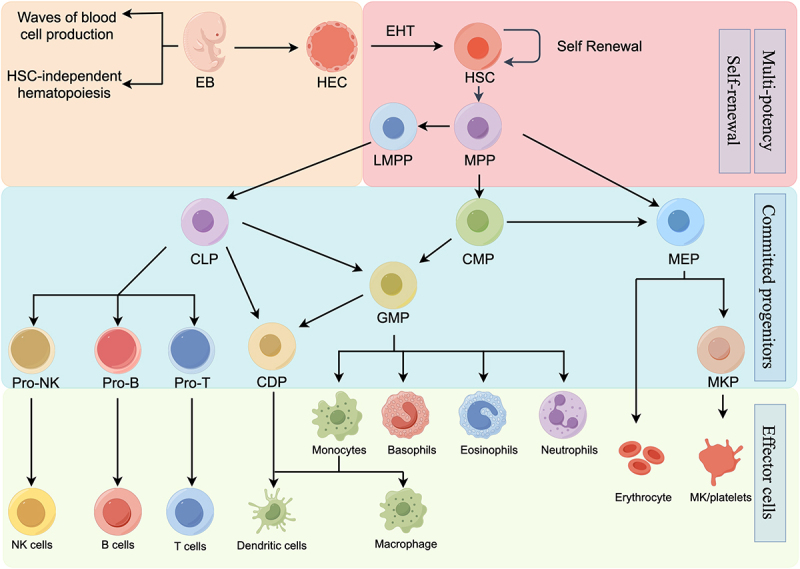
Hematopoietic differentiation hierarchy.

Hematopoiesis typically relies on HSCs as the primary source of hematopoiesis. However, it’s important to note that not all blood cells are exclusively generated by HSCs. Multiple waves of blood cell production originate from hemogenic endothelial cells in the yolk sac before the emergence of HSCs.^4,5^ These HSC-independent blood cells originate during the embryonic hematopoietic period and persist into the adult hematopoietic system. It’s worth mentioning that HSC-independent lymphocytes are present from the embryonic stage and continue into adulthood.^6^ In this article, we will focus exclusively on HSC-dependent hematopoiesis. (Figure 1 shows the hematopoietic differentiation hierarchy)

HSCs are at the apex of a hierarchy of multiple progenitor cell stages giving rise to all blood cell lineages.

EB, embryo; HEC, hemogenic endothelial cells; EHT, endothelial-to-hematopoietic transition; HSC, hematopoietic stem cell; MPP, multipotent progenitor; LMPP, lymphoid-primed MPP; CLP, common lymphoid progenitor; CMP, common myeloid progenitor; MEP, megakaryocyte-erythroid progenitor; GMP, granulocyte-monocyte progenitor; CDP, common dendritic progenitor; MKP, megakaryocyte progenitor; NK, natural killer cell.

HSCs originate during the embryonic period. In mammals, the earliest blood cells arise from the extra-embryonic yolk sac. During this stage, blood cells such as primitive erythrocytes are not generated by definitive HSCs, but rather, during the early stages of embryonic development, specifically at the mid-primitive streak stage (E7.0), the commitment of embryonic cells to hematopoietic fates begins in the proximal regions of the egg cylinder.^4,7,8^ Alongside the primitive hematopoiesis, a group of endothelial-like cells form a vascular-like structure around the blood cells.^9^ The appearance of blood cells and the presence of endothelial cells are closely temporally and spatially linked. Utilizing the differentiation of mouse embryonic stem cells into hematopoietic cells and endothelial cells, it has been confirmed that during embryonic body development, there exists a group of cells with distinct hematopoietic capabilities. These cells can be induced to differentiate into blast-like cells. These immature cells express a range of genes that are commonly shared between hematopoietic and endothelial lineages. When these immature cells are transferred to a culture medium under specific conditions, they can be induced to give rise to a proportion of colonies that contain hematopoietic precursors, as well as adherent cells exhibiting endothelial characteristics.^9^ These observations confirm that hematopoietic cells and endothelial cells share a common progenitor, known as the hemangioblast.^10^

Hemangioblasts in the yolk sac form blood islands, supporting the initial HSC-independent hematopoiesis, while also developing hematopoietic endothelium.^10^ Definitive HSC arises in a process termed endothelial-to-hematopoietic transition (EHT), in which specialized hemogenic endothelial cells (HECs) transform into hematopoietic cells.^11–13^ Additionally, the vicinity of the dorsal aortic floor within the Aorta-Gonad-Mesonephros (AGM) region was identified as the earliest anatomical site from which definitive HSCs could be isolated.^14,15^ From E10.5 onwards, HSCs begin to sprout from the ventral wall of the dorsal aorta, subsequently migrating into the vessel’s interior. Following this, significant morphological changes enable the newly specified HSCs to extravasate from the vascular wall into the bloodstream, facilitating their journey to the fetal liver.^16,17^ Once settled in the liver, HSCs initiate rapid expansion and differentiation into erythroid, myeloid, and lymphoid progenitors. As embryonic development unfolds, a portion of these HSCs subsequently migrate into the bone marrow for long-term habitation. HSCs in the bone marrow play a central role in establishing the hematopoietic system throughout the lifetime.

HSCs exhibit variations in self-renewal and differentiation capabilities, categorizing them into distinct subgroups such as long-term HSCs (LT-HSCs) and short-term HSCs (ST-HSCs). These subgroups subsequently generate multi-potent progenitors (MPPs), initiating a process of expansion that culminates in the formation of mature blood cells spanning diverse lineages. MPPs undergo differentiation, branching into common myeloid progenitors (CMPs) and common lymphoid progenitors (CLPs). The fate of CMP progenitors is determined by the presence of specific survival and differentiation factors, leading to the generation of progeny like granulocyte-monocyte progenitors (GMPs) and megakaryocyte-erythroid progenitors (MEPs). Conversely, CLP precursors embark on pathways that result in the differentiation into T cells, B cells, and natural killer (NK) cells, depending on the availability of distinct regulatory factors.

### 2.2. HSCs interact with BM niche

HSCs are present in bone marrow niche (BM niche) as quiescent or self-renewing cells. The concept of the bone marrow microenvironment, initially introduced by R. Schofield and referred to as the BM niche, comprises both cellular and non-cellular components.^18^ These components form an intricately interconnected molecular network that regulates the functional status of HSCs.

Sean J. Morrison’s team conditionally deleted various niche cell components to test which ones are essential for HSCs.^19,20^ They generated a floxed allele of stem cell factor (SCF) to conditionally delete SCF from candidate niche cells in mice. It turned out that when SCF expressed by endothelial cells (ECs) and leptin-receptor-expressing (LepR^+^) perivascular stromal cells were deleted, the number of HSCs decreased, and their function was impaired. However, in mice where SCF was deleted in osteoblasts and hematopoietic cells, no such effects were observed. Within the bone marrow vascular niche, ECs play a predominant role, raising the possibility that ECs might modulate HSC homeostasis.^21^ Using transgenic mice with GFP fluorescence, the authors demonstrated that ECs support the self-renewal of LT-HSCs through the secretion of specific paracrine growth factors, known as angiocrine factors, and this process relies on NOTCH signaling transmission.^22^ These angiocrine factors support the in vitro expansion of mouse CD34^−^Flt3^−^LSK hematopoietic stem and progenitor cells (HSPCs), exhibiting the phenotype and in vivo engraftment capability of LT-HSCs. Bone marrow-derived mesenchymal stem cells (BM-MSCs) are crucial components of the BM niche. A group of CD146^+^ mesenchymal stem and progenitor cells (MSPCs) was discovered in the BM niche, tightly clustered around arterioles and more loosely around sinusoidal blood vessels. When transplanted into recipient mice, these cells can form ectopic hematopoietic microenvironments, supporting host-derived HSCs.^23^ It is now widely accepted that mesenchymal stem cells (MSCs) are key components of the BM niche and major contributors to many known niche factors, such as CXCL12, SCF, and IL-7.^24^ Additionally, certain cellular components in the BM, such as adipocytes, are detrimental to HSCs.^25,26^ However, there has been a shift in this traditional view recently, with some studies suggesting that adipocytes may support the maintenance of HSCs.^27,28^ BM is innervated by autonomic nerve fibers, and the sympathetic nervous system (SNS) plays a crucial role in hematopoiesis. This regulatory mechanism affects the circadian rhythm fluctuations of CXCL12 in the BM microenvironment, thereby achieving molecular clock control transmitted from the central nervous system.^29^ Additionally, the progeny of HSCs themselves can also regulate HSC activity in a feedback loop. This includes megakaryocytes, neutrophils, regulatory T cells, and others, all of which contribute to the modulation of HSC function.^30^ (Table 1 lists the major cellular components and niche factors in the bone marrow microenvironment and their impact on hematopoiesis)

**Table 1.** Cellular and molecular constituents of the HSC niche.

Cell type	Soluble/membrane-bound niche factor	Cell phenotype	Effects on hematopoiesis
LepR^+^ stromal cells	SCF PTN^31^ CXCL12	Perivascular cells that express high levels of CXCL12 and SCF	Maintain HSCs^20^ and c-kit restricted hematopoietic progenitors^32^
Nes-GFP^+^ stromal cells	SCF CXCL12^29^	Nes-GFP^bright^ cells along with arterioles and Nes-GFP^dim^ cells associated with sinusoids	Maintain HSCs^33,34^ Regulate erythropoiesis^35^ HSCs mobilization and homing^36,37^ HSCs expansion^38^
NG2^+^ stromal cells	CXCL12	Periarteriolar labeled by NG2 pericyte marker^33^	Promote HSCs quiescence^39^
CAR cells	IL-6 SCF CXCL12	Cells that express high levels of CXCL12 and SCF	Sensing and integrating signals produced by pathogens for hematopoiesis under chronic inflammation^40^ HSC maintenance^41^
N-Cadherin^+^ stromal cells	SCF CXCL12	N-Cadherin^+^ cells in the endosteum of the trabecular bone region^42^	Preserve reserve HSCs^43^ Maintain functional HSCs^43^ Support hematopoietic regeneration post-myeloablation^43^
EC	SCF CXCL12 PTN^31^ E-/P-selectin ANGPTL2^44^	CD31^+^VE-Cadherin^+^VEGFR1^+^VEGFR2^+^ cells^45^	Maintain HSCs^20^ HSCs regeneration^31^ HSCs homing and engraftment^46^ Localization and specification of HSCs^1^ HSC stemness maintenance^44^
Osteoblasts	OPN G-CSF	Alkaline phosphatase^+^, osteocalcin^+^ and CD10^+^ bone-lining endosteal cells^47^	Negatively regulates HSC frequency^48^ Mediate HSC migration in and out of the BM^49^
Adipocytes	Adiponectin	Perilipin^+^ and Oil RedO^+^ cells, present throughout the bone marrow^26^	Sustain HSC survival^27^ HSCs regeneration^28^ Negative regulation of hematopoietic recovery^26,50^ and HSCs expansion^25^
Megakaryocytes	TPO TGF-β1 CXCL4	CD41^+^ cells, closely associated with sinusoids^51^	Maintenance of HSCs quiescence and promotion of HSC regeneration under stress condition^52,53^ HSCs differentiation^54^ Replenish hematopoietic cells^55^
Macrophages	TNF-α	CD45^+^F4/80^+^ cells and marked by Csf1r-GFP	Regulate erythropoiesis^56^
Regulatory T cells	IL-10	CD4^+^CD25^+^FOXP3^+^ cells	Regulate lympho-hematopoiesis^57^ Maintain HSCs and promote allo-HSCs engraftment^58^
Schwann cells	Integrin-β8	Myelinating Schwann cells express MBP and non-myelinating Schwann cells express GFAP	Maintenance of HSC hibernation^59,60^
SNS nerves	Noradrenaline Neuropeptide Y^61^	Synapsed on perivascular cells	HSPCs mobilization^62^ HSCs regeneration^61^ HSCs hibernation^61^

Various cell types have been implicated in regulating hematopoietic stem cell (HSC) activity. They contribute to HSC regulation indirectly or directly by synthesizing niche factors in the form of cell-bound or secreted molecules.

HSCs, hematopoietic stem cells; LepR+ stromal cells, leptin-receptor-expressing stromal cells; SCF, stem cell factor; PTN, pleiotrophin; CXCL12, CXC-chemokine ligand 12; Nes-GFP+ stromal cells, Nestin-expressing-GFP+ stromal cells; CAR cells, CXCL12 abundant reticular cells; IL-6, interleukin-6; EC, endothelial cells; ANGPTL2, angiopoietin-like proteins 2; VEGFR, vascular endothelial growth factor receptor; OPN, osteopontin; G-CSF, granulocyte-colony stimulating factor; TPO, thrombopoietin; TGF-β1, transforming growth factor-β1; CXCL4, CXC-chemokine ligand 4; TNF-α, tumor necrosis factor-α; MBP, myelin basic protein; GFAP, glial fibrillary acidic protein; SNS, sympathetic nervous system.

A recent concept suggests that the bone marrow microenvironment, essential for the survival of HSCs, responds to changes in the host’s gut microbiota, thereby regulating the fate of HSCs. This regulatory effect may be associated with changes in metabolic products, aging of the BM niche, alterations in inflammatory signals, and shifts in cytokines.^63^ Collectively, these factors impact the proliferation, self-renewal, and differentiation fate of HSCs.

## 3. Gut microbiota and hematopoiesis

For a long time, the connection between the gastrointestinal system and the hematopoietic system has been challenging to establish due to their lack of inherent anatomical proximity and distinct physiological functions. However, this perception is not accurate. We believe that the gut-blood axis represents the pathway through which microbes, by regulating immune and metabolic pathways, impact the host and reshape its hematopoietic function.^64,65^ The introduction of this concept marked a significant advancement.

### 3.1. Gut microbiota correlates with hematopoiesis during homeostasis

#### 3.1.1. Innate immunity

For HSCs, the presence of the gut microbiota is essential, as it has been demonstrated to contribute to the stable maintenance of HSCs in the bone marrow.^66,67^ In STAT1 gene knockout mice, a phenotype similar to hematopoietic impairment induced by broad-spectrum antibiotic treatment was observed. This suggests that the microbial community maintains stable hematopoiesis through the activation of the STAT1 signaling pathway.^68^ Early microbial colonization in newborns may potentially favor rapid hematopoiesis in newborns, at least in relation to the swift generation of neutrophils to cope with infections during the neonatal period.^69,70^

The impact of the gut microbiota extends beyond maintaining HSCs, demonstrating its influence on changes in HSCs output patterns. To maintain an intrinsic immune homeostatic environment, the host continuously recognizes foreign antigens and responds to them. Pathogen-associated molecular patterns (PAMPs), relatively conserved recognition domains carried on the surface of the gastrointestinal microbiota, bind to pattern recognition receptors (PRRs) on host immune cells. Among these, the Toll-like receptors (TLRs) family, expressed on the cell membrane and intracellularly, plays a pivotal role in mediating the recognition of various immune antigens, including PAMPs, lipopolysaccharides (LPS), and viral RNAs.^71,72^ The Lactobacillus rhamnosus GG strain, as a probiotic, is widely used in food additives. Its characteristic feature lies in possessing pili, which endows it with immunomodulatory capabilities, enabling it to elicit targeted responses in human immune-related cells. In general, the pili of Lactobacillus rhamnosus GG strain can activate TLR2-dependent signaling in cells, triggering subsequent NF-κB inflammatory signaling pathway. Additionally, it can modulate the production of pro-inflammatory cytokines (TNF-α, IL-6, IL-10, and IL-12) in human monocyte-derived dendritic cells.^73^ The intestinal microbiota regulates hematopoiesis of monocytes through microbial products, directly impacting the lifespan of monocytes. Depletion of microbiota due to antibiotic consumption increases the number of Ly6C^high^ monocytes in peripheral blood. In antibiotic-treated mice, monocyte survival is enhanced through the nucleotide-binding domain 1 (NOD1) pathway. The apoptosis rate of Ly6C^High^ monocytes from NOD1^−^/^−^ mice is higher compared to wild-type monocytes.^74^

**Figure 2.**
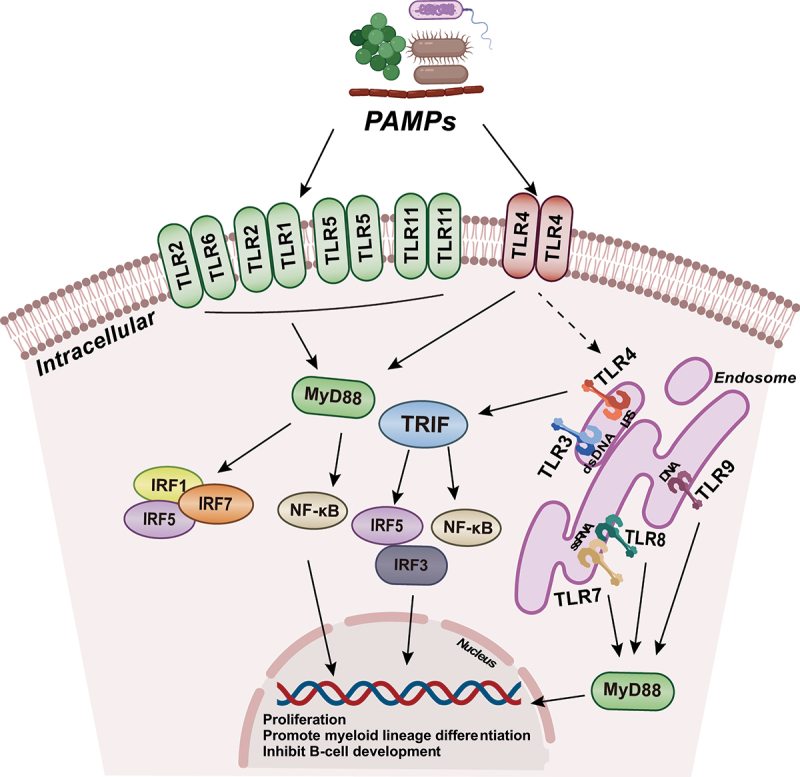
Toll-like receptor signaling in HSCs proliferation and differentiation.

Through TLRs, HSCs sense the microbial flora, and the induction of specific myeloid cells can be achieved in both humans and mice through different TLR ligands.^75–77^ TLR1/2 agonist PAM_3_CSK_4_ can favor their differentiation into mature functional myeloid cells belonging either to the monocytic or dendritic cell (DC) lineages.^78^ TLR agonists can profoundly inhibit B-cell development bias meanwhile in favor of myeloid development by targeting HSPCs. Further exploration was made to determine that PAM_3_CSK_4_ significantly upregulated the expressions of the GATA-1, C/EBPα and PU.1 transcript in HSCs, which shifts the transcriptional network toward the myeloid path.^78^ BM-MSCs, one of the components of the BM niche, also express TLRs. Stimulation of TLR4 with LPS enhances the ability of BM-MSCs to promote myeloid lineage differentiation of HSPCs as well as the proliferation capacity of HSCs.^79,80^ Treating adult BM HSCs with a TLR7/8 specific ligand can induce TLR7/8 signaling, leading to the differentiation of CD34^+^ progenitor cells into CD11c^+^CD14^+^ myeloid dendritic cells.^75^ In mice lacking gut microbiota, there is a significant decrease in phagocytes, monocytes, and neutrophils in the spleen and bone marrow. Phagocytes in the spleen also exhibit lower proliferative capacity. Furthermore, germ-free (GF) mice show a reduced proportion of GMPs in the bone marrow.^81^ Myeloid development originates from HSCs in the bone marrow, and this differentiation deficiency may be associated with the MyD88/TICAM signaling pathways mediating by gut microbiota.^76,82^ These observations confirm that the absence of gut microbiota affects myeloid development (Figure 2 shows the relationship between TLRs family-mediated intestinal flora and HSC function). In addition, antibiotics cause multilineage alterations in murine hematopoiesis, with marked suppression of multipotent progenitors.^68^ After infection with Escherichia coli, Lin^−^c-kit^+^Sca-1^+^ cells in Balb/c mice bone marrow significantly increased, and the number of granulocyte/macrophage colony-forming units (CFU-GM) also increased.^83^ Although this experiment does not directly demonstrate the impact of gut-colonizing microbes on mouse bone marrow HSCs, it still illustrates the microbiota’s capacity to shape hematopoiesis. The regulation of megakaryocytic lineage development by the gut microbiota remains undetermined, although some studies suggest that the absence of gut microbiota can lead to changes in the morphology and size of adipocytes in the bone marrow. Fatty acids secreted by adipocytes are believed to aid in the maturation of megakaryocytes, thereby indirectly regulating megakaryocyte maturation.^84^ Some research teams also suggest that the gut microbiota, by secreting propionate, a type of SCFA, reduces the number and frequency of MEP cells in the mouse bone marrow, leading to a subsequent decrease in the production of megakaryocytes. However, these gut microbiota metabolites can induce an increase in megakaryocyte maturation and platelet production.^85^ Research on the role of the gut microbiota in megakaryocytic lineage development remains lacking, requiring further in-depth exploration in future studies.

TLR1, TLR2, TLR4, TLR5, TLR6 and TLR11 are located on the cell surface, while TLR3, TLR7, TLR8 and TLR9 are located in the endosome/lysosome. TLR activation initiates a MyD88/TRIF-dependent signaling cascade, which can activate NF-κB and IRF, resulting in the proliferation and biased-differentiation of HSC.

HSCs, hematopoietic stem cells; TLR, Toll-like receptors; IRF, interferon-regulatory factors; PAMPs, Pathogen-associated molecular patterns.

#### 3.1.2. Adaptive immunity

The aging process is accompanied by significant changes in hematopoietic function, characterized by a decline in the self-renewal capacity of HSCs and a shift in lineage differentiation from the lymphoid lineage toward the myeloid lineage. In the early stages of aging, a progressive loss of MPP4, which with lymphoid commitment, can be observed. Transplanting MPP4 from aged mice into the bone marrow of young mice, this loss cannot be rescued by a younger bone marrow microenvironment, indicating that the biased differentiation potential of MPP4 stems from cell-autonomous changes in the MPP compartment with aging.^86^ However, this alteration is reversed in GF mice, where the aging-induced shift from lymphocytes to granulocytes is largely abrogated. Compared to aged SPF mice, aged GF mice show a significant increase in the percentage of B cells in the BM and peripheral blood, with a slight decrease in the percentage of granulocytes. In addition, the absolute numbers of MPP4 with lymphoid commitment and B cell precursors decline within aged mice, while in GF mice, this trend is reversed. With aging, the percentages and absolute numbers of CLP and B cell precursors significantly increase in GF mice.^87^ These results suggest that the gut microbiota exerts a negative regulatory inhibitory effect on the generation of lymphoid cells, especially B cells, during the aging process.

The gut microbiota also regulates the progeny of hematopoietic cells and wields the power to mold the host’s immune milieu. The influence of the gut microbiota on the host’s immune system is not limited to the mucosal surface of the intestine but can extend to systemic compartments. As we comprehend, Bacteroides fragilis, an inhabitant of the gastrointestinal tract, harbors zwitterionic polysaccharides (ZPSs) that orchestrate the proliferation of host CD4^+^ T cells. A pivotal immunogenic component within ZPSs, designated as polysaccharide A (PSA), undergoes modification within antigen-presenting cells (APCs) and, upon presentation via major histocompatibility complex class II (MHC II) molecules, incites the activation of CD4^+^ T cells. It is worth underscoring that in the spleens of GF mice, the CD4^+^ T cell proportion experiences a significant reduction compared to conventionally raised mice, signifying an impairment in T cell development. Nonetheless, following the reintroduction of Bacteroides fragilis, the CD4^+^ T cell ratio is reinstated.^88^ Similarly, the developmental deficiency of Foxp3^+^ Treg cells is likewise rectified upon exposure to PSA.^89,90^ The identical immunomodulatory capability has been consistently verified across different commensal gut bacteria in mouse models.^91–93^ Similarly, recent clinical studies have demonstrated that higher diversity in the host gut microbiota α-diversity predicts better clinical outcomes. This beneficial regulation arises from the gut microbiota metabolites’ control over adaptive immunity. Higher concentrations of riboflavin and the molecule 4-hydroxy-3-methyl-but-2-enyl pyrophosphate (HMBPP) activate innate-like mucosal-associated invariant T cells (MAIT), especially Vδ2 T cells. These donor-derived MAIT and Vδ2 cells may protect against acute GVHD, although the mechanisms underlying these patient outcome variations remain unclear.^94^ Therefore, gut microbiota seems to play a role in fostering the development of the host’s immune system.

The above evidence indicates that the gut microbiota regulates HSCs differentiation within the bone marrow, leading to direct changes in myeloid and lymphoid lineage commitment, thereby modulating the development of blood cells and participating in immune cell differentiation. With such pervasive effects, researchers have gained further confidence in the microbiota’s role not only in the regulation of hematopoiesis but also in innate and adaptive immunity.

### 3.2. Gut microbiota in hematopoiesis under stress conditions

Current evidence suggests that the gut microbiota can regulate lymphocyte function in secondary immune organs.^95–97^ Moreover, under various disease conditions or hematopoietic stress, the gut microbiota can also modulate the function and fate of HSCs in primary immune organs.

#### 3.2.1. Infection

The elimination of gut microbiota imposes restrictions on the generation of multi-lineage cells in peripheral blood and bone marrow, underscoring the indispensable role of gut microbiota in hematopoiesis.^68^ During the host infection process, HSCs can be activated through both direct (infection or induction) and indirect (inflammation or changes in the ecological niche) mechanisms. As previously mentioned, the gut microbiota influences HSCs in the bone marrow, activating specific receptors and causing differentiation bias. This timely response provides essential support to the host’s innate and adaptive immune systems. When facing pathogens, the gut microbiota enhances the host’s ability to respond to infections by controlling the production of innate immune cells derived from HSCs.^81^ When GF mice and SPF mice were intravenously injected with equal doses of Listeria monocytogenes during the primary immune response, GF mice exhibited higher bacterial titers in the liver and spleen, shorter survival time, and higher mortality rates. Subsequently, during secondary immunization with the same procedure, bacterial titers in the liver and spleen of GF mice were barely detectable.^98^ This indicates that lack of microbiota increases susceptibility to primary infection but enhances resistance against secondary infection. In the spleen of GF mice during secondary immunization, infiltration of neutrophils and macrophages was reduced, while the number of CD8^+^ memory T cells dramatically increased.^98^ This suggests that the lack of microbiota reduces innate immune response and enhances adaptive immunity. It primarily impairs early recruitment and activation of granulocytes, leading to increased memory T-cell responses. During infection, the gut microbiota influences the hematopoiesis. Infections trigger an immune response, including an inflammatory reaction. This inflammatory state has adverse effects on HSCs and the hematopoietic process. Specifically, infection-induced inflammation can result in damage to HSCs, leading to a decline in hematopoietic function and even causing issues such as anemia. The gut microbiota may play a role in mitigating or exacerbating hematopoietic problems caused by infection by modulating the immune system’s response and the degree of inflammation. Commensal gut bacteria provide complex molecular signals, including PAMPs, actively promoting the proliferation and differentiation of myeloid cells, contributing to the early detection and control of systemic bacterial infections.^81,99^

#### 3.2.2. Anemia

Anemia is one of the most common disorders of the hematopoietic system, with diverse causes, primarily centered around depletion of hematopoietic raw materials, utilization disorders, and loss of blood cells. Iron utilization is crucial for red blood cells as it is a vital component of hemoglobin. Systemic iron deficiency or utilization disorders can result in impaired red blood cell production, leading to anemia. The acquisition of iron in mammals is regulated by duodenal enterocytes through a comprehensive systemic and local regulatory system. Consequently, maintaining a balanced gut microbiota is crucial for ensuring systemic iron equilibrium. When exposed to diets with restricted iron content, noticeable distinctions in peripheral blood cell counts were observed between GF mice and SPF mice. SPF mice exhibited lower hemoglobin levels, suggesting a more severe state of anemia.^100^ This indicates that the presence of gut commensal bacteria might competitively acquire iron, resulting in a more pronounced anemic condition in SPF mice subjected to restricted iron intake. Intestinal iron absorption is primarily influenced by the transcription factor hypoxia-inducible factor-2α (HIF-2α), which acts on iron transport proteins. Subsequent analysis of organics extracted from the intestine confirmed that these are metabolic byproducts generated by gut commensal bacteria. These byproducts inhibit the expression and activity of intestinal HIF-2α, restricting the absorption of iron in the small intestine, ultimately leading to impaired erythropoiesis.^100^ Moreover, the gut microbiota responds to hematopoietic stress by modulating the systemic availability of iron.^101^ The phagocytosis of red blood cells under steady-state conditions is primarily mediated by macrophages and mainly occurs in the spleen and bone marrow. This process provides iron elements for red blood cell regeneration, and intracellular iron balance can directly influence HSCs function. Treatment with 5-fluorouracil (5FU) enhances macrophage efficiency in clearing red blood cells. However, in mice treated with ABX and subsequently induced with bone marrow injury using 5FU, the observation of bone marrow regeneration reveals a slower rate of red blood cell clearance. Simultaneously, the mice exhibit a delayed multi-lineage hematopoietic reconstruction, an increased number of HSCs in the bone marrow, more active cell cycling, and a blockade of HSCs differentiation.^101^ Under steady-state conditions, bone marrow phagocytic activity is minimal. However, under 5FU-induced stress, the early stages of bone marrow regeneration trigger a robust red blood cell phagocytic response, which is significantly reduced in GF conditions. These results indicate that the gut microbiota regulates the stress-induced phagocytic activity of bone marrow macrophages on red blood cells. The stress-induced recycling of red blood cells in the bone marrow may contribute to hematopoietic regeneration, influencing HSCs self-renewal by modulating the availability of iron.

#### 3.2.3. Hematologic malignancy

Chemotherapy is the cornerstone of hematologic malignancy treatment but can lead to severe bone marrow damage. Adjusting the composition of the gut microbiota can help protect against chemotherapy-induced immune suppression. Cyclophosphamide (CTX) treatment leads to a substantial reduction in CMP, MEP, and CLP levels. Administration of specific drugs inhibits the CTX-induced increase in Proteobacteria and Campilobacterota, preserving the predominant bacterial levels of Firmicutes and Bacteroidota. This effectively reverses the decline in CLP induced by CTX and significantly enhances the quantity of HSPCs. However, in mice where the gut microbiota has been depleted, this protective effect is no longer observed.^102^ This suggests that the gut microbiota plays a critical role in promoting bone marrow hematopoiesis and restoring immune function under the hematopoietic stress induced by bone marrow injury.

Allogeneic hematopoietic stem cell transplantation (allo-HSCT) and autologous hematopoietic stem cell transplantation (auto-HSCT) are other effective approaches for treating hematologic malignancy. The prevailing consensus suggests that patients undergoing allo-HSCT may experience substantial changes in the gut microbiota, potentially leading to diverse health challenges.^103,104^ Following HSCT, there is a complex bidirectional interaction between intestinal inflammation and immune responses, with the gut microbiota acting as a bridge. For instance, the activity of the gut microbiota’s metabolites, such as short-chain fatty acids (SCFAs), correlates with the severity of post-transplant GVHD.^103,105^ In severe GVHD patients, we observed a significant decrease in SCFAs levels, indicating a correlation between altered gut microbiota metabolism and the severity of GVHD. Analysis of gut microbiota in patients undergoing auto-HSCT in a multicenter clinical study revealed a significant decrease in α-diversity during the peri transplantation period, suggesting a disruption in the patients’ gut microbiota homeostasis.^106^ This disrupted homeostasis is accompanied by specific changes in microbial composition, where certain facultative anaerobes gradually become dominant microbiota post-transplantation, including Streptococcus and Enterococcus. The shift in dominant microbiota and changes in α-diversity serve as one of the reasons for the differences in patient outcomes. Children with high abundance of Lactobacillaceae are more prone to severe GVHD and have a higher mortality rate following allo-HSCT. Conversely, high abundance of Clostridiales, represented by Ruminococcaceae and Lachnospiraceae, alleviates gut microbiota dysbiosis in these allo-HSCT pediatric patients. This subset of patients experiences significantly reduced incidence and severity of GVHD and achieves better clinical survival.^107^

The improvement in clinical outcomes is believed to be closely associated with the recovery of the adaptive immune system. Patients with higher peri engraftment α-diversity exhibit higher absolute numbers of peripheral blood CD4^+^ and CD8^+^ T cells at 3- and 6-months post-transplantation. Further taxonomic differences were investigated, revealing that patients with a high abundance of the Erysipelotrichaceae class have faster T cell recovery rates and lower post-transplant complications.^108^ Conversely, enrichment of Clostridiales accelerates NK and B cell reconstitution,^107^ similarly reducing the risk of GVHD and prolonging patient survival. However, Enterococcus and Staphylococcus are associated with an increased risk of infections and higher mortality, a conclusion consistent with previous multicenter clinical studies.^106,108^ Patients with higher microbial diversity also exhibit faster recovery of the innate immune system following HSCT, characterized by rapid reconstitution of neutrophils and monocytes in peripheral blood.^109^ These findings suggest that changes in microbial diversity and abundance regulate the pace of adaptive and innate immune system recovery after HSCT.

#### 3.2.4. Metabolic alterations

The gut microbiota participates in the breakdown and absorption of food, thereby influencing the host’s nutritional status. Adequate nutrition is essential for supporting the hematopoietic process, with the gut microbiota playing a key role in promoting hematopoiesis by maintaining nutritional balance. Additionally, the gut microbiota produces bioactive metabolites through food breakdown, which effectively address hematopoietic stress. In allergic respiratory diseases, mice fed with different dietary fiber contents exhibit variations in the diversity and abundance of gut microbiota, leading to differences in SCFA levels. This alteration in gut microbiota composition is associated with changes in bone marrow hematopoietic function. Specifically, it manifests as enhanced generation of macrophage and DC precursors, followed by the seeding of the lungs by DCs with high phagocytic capacity but an impaired ability to promote type 2 T helper (Th2) cell effector function.^65^ This change mitigates the severity of lung damage. Obesity stands as one of the prominent global public health challenges today, closely linked with metabolic syndrome and involving a range of physiological changes, such as abnormal distribution and functionality of adipose tissue, insulin resistance, and inflammatory responses. In terms of hematopoietic function, the gut microbiota mediates the bias in obesity-induced HSCs differentiation. In obese mice, alterations occur in the composition of Gram-positive bacteria within the microbiome, characterized by elevated levels of Verrucomicrobia, Actinobacteria, and Proteobacteria. Additionally, there is a trend of increased absolute numbers of bone marrow LSK cells, suggesting a propensity toward generating CMP during LSK differentiation, while the quantity of CLP gradually decreases. Antibiotic treatment mitigates these shifts in gut microbiota observed in obese mice, thereby ameliorating the bias in HSCs differentiation.^110^ In essence, obesity affects the differentiation pattern of the hematopoietic lineage toward the myeloid lineage at the expense of the lymphoid lineage through the gut microbiota as an intermediary factor.

### 3.3. Regulation of BM niche by gut microbiota

The hematopoietic process, orchestrated by HSCs, is intricately connected to a multitude of factors within the body, shaping a dynamic network of regulatory influences. Within this framework, the BM niche emerges as a pivotal provider of signals and support crucial for the proper differentiation and proliferation of HSCs. Remarkably, the gut microbiota appears to exert its influence on BM niche functionality through diverse mechanisms, thereby reshaping the behavior of HSCs. This interplay not only enhances our comprehension of the intricate regulatory mechanisms governing hematopoiesis but also unveils novel avenues for future medical exploration and therapeutic interventions.

Cells are equipped with PRRs, such as TLRs, NOD, and leucine-rich repeat-containing protein receptors (NLRs). Notably, NOD1 and NOD2 are responsible for recognizing peptidoglycan (PGN), a component derived from the cell walls of both Gram-positive and Gram-negative bacteria.^111^ NOD1 and NOD2 are primarily expressed in the cytosol. In contrast to the TLR family, which primarily recognizes PAMPs at the cell surface, NOD1/2 are involved in the intracellular sensing of both pathogenic and commensal bacteria.^111,112^ Lower levels of NOD1 ligands were detected in the serum of GF mice or SPF mice treated with antibiotics. Following supplementation with NOD1 ligands, there was an increase in the proportion of LSK cells in the bone marrow of mice. Further analysis revealed an elevation in multiple lineage levels, including LT-HSC, ST-HSC, MPP, CLP, and CMP. Importantly, this phenomenon was not observed in NOD1^−^/^−^ deficient mice, providing evidence for the regulatory role of NOD1 signaling in steady-state hematopoiesis.^113^ However, the observed alterations in HSPCs regulated by NOD1 ligands could not be verified in vitro studies, implying the involvement of an additional regulatory factor. Ultimately, upon isolating MSC cells from the BM niche, the researchers confirmed that the stimulation of BM-derived MSCs with NOD1 ligands resulted in the secretion of cytokines, including IL-3, IL-7, Flt3L, SCF, and thrombopoietin (TPO). The NOD1 ligand from the microbiota stimulates BM-derived MSCs, influencing the production of hematopoietic cytokines in vivo. These cytokines are responsible for inducing HSPC proliferation.^113^ The lactic acid produced from the gut microbiota stimulates leptin receptor-expressing (LepR^+^) MSCs to generate SCF, inducing elevated levels of cytokines in the bone marrow niche environment. This, in turn, promotes HSCs proliferation and erythropoiesis.^114^

BM-MSCs are not the sole factor responsible for the alteration of host hematopoiesis under the influence of the gut microbiota. When comparing GF mice with conventionally raised mice, although the levels of inflammatory cytokines in the peripheral blood are undetectable in both groups, GF mice exhibit even lower levels of inflammatory cytokines, represented by Tumor necrosis factor-α (TNF-α), IL-1β, and IL-6, in the bone marrow. Upon the simultaneous blockade of these three cytokines, there is a reduction in the numbers of HPC, MPP2, and myeloid lineage cells in the bone marrow. This confirms that the gut microbiota induces inflammatory cytokines in the bone marrow, maintaining the baseline levels of HSCs under steady-state conditions, despite the low levels of these cytokines. Bacterial DNA, primarily from the Proteobacteria phylum, enters the bone marrow through the circulation and is perceived by CX3CR1^+^ mononuclear cells (MNC), which generate inflammatory cytokines in the bone marrow under steady-state conditions.^67^ In summary, the gut microbiota is sensed by mononuclear cells in the bone marrow, inducing the production of inflammatory cytokines by the latter, thereby maintaining the baseline levels of HSCs under steady-state conditions.

The gut microbiota achieves a shift in HSCs’ output mode by influencing the BM niche. Induced by a high-fat diet, a significant alteration in the gut microbiota diversity in the cecum and ileum of mice was observed, characterized by increased levels of Verrucomicrobia, Actinobacteria, and Proteobacteria. Concurrently, there was an increase in the number of MPP and CMP in the bone marrow, while CLP decreased. When the gut microbiota of high-fat diet-fed mice was transferred to conventionally fed mice, similar changes in blood cells in the bone marrow were observed. Subsequent investigations revealed that the alteration in gut microbiota induced by a high-fat diet led to an increase in PPARα and PPARγ2 mRNA levels in the BM niche, and changes in bone matrix components were even observed in long bones, characterized by a decrease in osteoblasts and an increase in adipocyte numbers.^110^

## 4. Targeting gut microbiota to regulate hematopoiesis

Gut microbiota matters in orchestrating the function of various organs and systems throughout the body, sparking considerable interest in its intricate interplay with the hematopoietic system. The targeted modulation of hematopoiesis by the gut microbiota has emerged as a burgeoning area of research. This not only holds the potential to deepen our comprehension of hematopoietic mechanisms but also opens up new avenues for devising innovative intervention strategies and therapeutic approaches. In this section, we will explore interventions aimed at the precise control of hematopoiesis by targeting the gut microbiota and charting new courses for future research in the treatment of hematopoietic-related disorders.

### 4.1. Structure of gut microbiota

The structure of the gut microbiota, including α-diversity (biodiversity and richness within individuals) and β-diversity (bacterial community composition between individuals), has been described as a major environmental factor capable of regulating various biological processes. Disruption of microbiota structure is also a contributing factor to several diseases. Fecal microbiota transplantation (FMT) involves introducing a fecal microbiota preparation, containing microbiota from the distal intestine, into the gastrointestinal tract of patients with disrupted intestinal function or dysbiosis.^115^ Currently, FMT is most widely used clinically to treat recurrent Clostridioides difficile infections, with an average cure rate of up to 90%.^116^ With advancing research on the gut microbiota, some studies have highlighted the significant potential of FMT in improving host hematopoiesis.

With aging, the functionality of HSCs undergoes inevitable alterations. Beyond lineage-biased differentiation, aging precipitates a marked reduction in HSC regeneration and engraftment efficacy following transplantation. In more severe instances, this may coincide with the onset of hematologic malignancies, representing a distinctive manifestation of HSC dysfunction. Through FMT, wherein the gut microbiota from young mice is transplanted into aging mice, noteworthy outcomes emerge. Aging mice subjected to this intervention exhibit heightened peripheral blood counts of white blood cells (WBC), B cells, and CD8^+^ T cells, while experiencing a reduction in neutrophils within the myeloid lineage. Meanwhile, the BM demonstrates an augmentation in direct HSPCs and CLP. These findings suggest that FMT intervention contributes to the restoration of impaired lymphoid hematopoiesis in aging mice, although no observed improvements in CD4^+^ T cells. After secondary bone marrow transplantation, aging mice recipients of FMT from their younger counterparts display enhanced engraftment capabilities of HSCs within the bone marrow. These results underscore the efficacy of FMT in rectifying hematopoietic defects associated with aging. Furthermore, this restorative effect is correlated with an increased abundance of bacterial genera, including Butyricicoccus, Lachnospiraceae, and Clostridium, within the gut microbiota of the recipient mice. Notably, the deliberate supplementation of these identified microbial populations also demonstrates the capacity to improve the self-renewal ability of HSCs and restore lymphoid differentiative potential.^117^

FMT has an impact on clinical outcomes in patients undergoing allo-HSCT. In a Randomized Double-Blind Phase II Trial, patients who received oral FMT experienced alleviation of all-cause infection rate. While statistical differences were not observed in this clinical trial, it still acknowledges the positive value of FMT in the context of HSCT.^118,119^ Van den Brink’s lab explored the value of the intestinal microbiota in recipient hematopoietic system reconstruction after bone marrow transplantation. They found that in mice lacking intestinal microbiota, there is impaired hematopoietic system reconstruction after bone marrow transplantation. This is characterized by reduced peripheral blood lymphocytes, monocytes, and neutrophils, as well as lower bone marrow cellularity. Flora depletion impaired myeloid and lymphoid differentiation while largely sparing the stem and progenitor cell compartments and erythroid lineage. Surprisingly, the hematopoietic disruption caused by the lack of intestinal microbiota is attributed to severe impairment in the host’s carbohydrate energy absorption. This indicates that the intestinal microbiota alters the host’s energy metabolism, increases visceral fat accumulation, and promotes post-transplant hematopoietic reconstruction. This fundamentally enhances the efficiency of hematopoietic system reconstruction post-transplantation.^120^ The intestinal mucosal epithelial cells and microbiota establish a balanced state, thereby promoting optimal absorption of nutrients and resistance to infection. Pediatric patients undergoing HSCs transplantation experience frequent and varied changes in intestinal microbiota diversity and abundance. Disruption of this microbial balance leads to host nutritional imbalance, triggering severe infections including viremia.^121,122^ Probiotics are “live microorganisms that confer health benefits on the host when administered in adequate amounts”.^123^ Orally administered lactate-producing bacteria to mice altered the colonization of dominant bacteria in the intestines, accelerating hematopoiesis and erythropoiesis in the BM.^114^ In summary, restoring intestinal homeostasis through FMT significantly shapes the host’s hematopoiesis.

### 4.2. Inflammatory signal

Inflammatory signals are essential during the emergence, specification, and steady-state expansion processes of HSCs. The formation of definitive HSCs is established on the foundation of HECs, and TNF-α is a powerful proinflammatory cytokine that acts on vascular formation. TNF receptors regulate vascular homeostasis in zebrafish through a caspase-8, caspase-2, and P53 apoptotic program that bypasses caspase-3. It is reasonable to infer that TNF-α is also indispensable for the formation of HSCs. In the zebrafish embryo model, Espín-Palazón et al. demonstrated the essential role of TNF-α signaling stimulation, specifically through TNFR2, in the process of definitive HSC formation. Subsequent cascade signals activate the Jag1α/Notch1α signaling pathway in HECs. This Notch signaling is well-recognized as a hematopoietic regulatory signal in the complex regulation of HSC emergence by the Notch signaling pathway. Simultaneously, TNF-α activates the recruitment of adaptor proteins, triggering the activation of the transcription factor NF-κB. Through these two pro-inflammatory signals, definitive HSC formation is induced.^124^ Inflammatory signals parallel the maturation process of HSCs. HSCs mature in the AGM region and migrate to the liver. This migration is accompanied by increased expression of interferon-α (IFN-α) and IFN-γ. The IFN-α/Jak/Stat1 pathway plays a role in the maturation of HSC function.^125^ Additionally, IFN-α signaling-induced HSC exhaustion can be alleviated after blocking this signal.^126^ Therefore, it can be observed that a complex network of inflammatory signaling pathways collectively contributes to the formation and maturation of hematopoietic function.

While we have discussed the significant value of inflammatory factors in the development and maturation stages of HSCs, the intricate relationships involved in this process remain incompletely understood. For instance, TNF-α seems to exhibit contrasting effects in different contexts. TNF-α plays a role in coordinating the in vivo survival of HSCs and myeloid lineage development.^127^ Additionally, in the context of HSCT, bone marrow-derived CD8^+^/TCR^−^ graft facilitating cells (FC) promote HSCs proliferation by producing TNF-α, while simultaneously inhibiting HSCs apoptosis.^128^ Granulocytes in the bone marrow express the membrane-bound form of TNFα, stimulating the proliferation of EC in HSCT recipient mice.^129^ Since EC secretion of cytokines is beneficial for hematopoietic recovery, this may provide a positive stimulatory growth signal for HSCs. Another perspective suggests that TNFα acts as an inhibitory factor for hematopoietic stem cells. In vitro treatment of HSCs with TNF-α leads to a decrease in their post-transplantation proliferative and differentiation abilities.^130,131^ Early increases in TNF-α after HSCT restrict HSCs engraftment, while blockade of TNF-α enhances the reconstitution capacity of HSCs.^132^ The contrasting observations mentioned above suggest that the response of HSCs to TNF-α signaling may rely on the specific local environment in which the HSCs reside. Furthermore, the dosage and duration of exposure to TNF-α are critical factors to consider. Low doses of TNF-α may be necessary for the proper functioning of HSCs, while high doses of TNF-α over an extended period can result in the depletion of HSCs function.

Some of the inflammatory signals regulating hematopoiesis are elicited by the gut microbiota. With the increasing diversity of the gut microbiota, there is a sustained increase in GMP, neutrophils and monocytes in the bone marrow. Injecting serum from conventional mice into GF mice can improve myeloid lineage generation in GF mice. MyD88/TICAM1 deficiency disrupts the gut microbiota’s regulation of myeloid hematopoiesis. This is because gut microbiota-derived highly heat-stable proteins circulate in the plasma, forming a positive myelopoiesis stimulus through the MyD88/TICAM1 inflammatory signaling pathway.^82^ In another study using GF mice, PRRs were artificially activated by PAMPs-like stimuli. Oral administration of heat-killed Escherichia coli strain Nissle promoted the generation of granulocytes and monocytes in the bone marrow of GF mice. This suggests that the gut microbiota utilizes inflammatory signaling pathways to regulate myelopoiesis in the absence of specific microbial components.^81^ Perinatal antibiotic administration restricted the colonization and total abundance of gut microbiota in neonatal mice, resulting in significantly decreased numbers of neutrophils and GMPs in both bone marrow and peripheral blood, through a TLR4- and MyD88-dependent pathway. Loss of activation in the TLR4 and MyD88 signaling pathways resulted in the inability to induce the production of IL17-producing group 3 innate lymphoid cells (ILC), along with decreased plasma levels of G-CSF, which attenuated postnatal granulocytosis.^133^ The gut microbiota continuously produces and secretes low levels of TLR ligands and NOD ligands, which act locally on the intestinal surface or enter the bloodstream, determining the differentiation bias of monocyte-derived macrophages and DCs.^134^

### 4.3. Microbiota-derived metabolites

The metabolic products of the gut microbiota arise from bacterial synthesis using dietary components, modification of host-generated metabolites by intestinal bacteria, and de novo synthesis of metabolites by the gut microbiota. Extensive research has affirmed that the gut microbiota regulates host responses by producing a diverse array of active metabolites. Consequently, targeting microbial metabolism has emerged as the most feasible approach to regulation.^135^ Iron is a crucial trace element for mammals, and competition for iron in the gut is vital for maintaining the indigenous microbial community and host health. Through high-throughput screening of microbial metabolites, the authors discovered that the gut microbiota produces metabolites that can inhibit hypoxia-inducible factor-2α (HIF-2α), increase ferritin levels, and consequently reduce host absorption of intestinal iron. 1,3-diaminopropane (DAP) and reuterin are inhibitors of HIF-2α, effectively alleviating systemic iron overload. This work provides evidence for the critical role of gut microbiota metabolites in systemic iron balance.^100^

**Figure 3.**
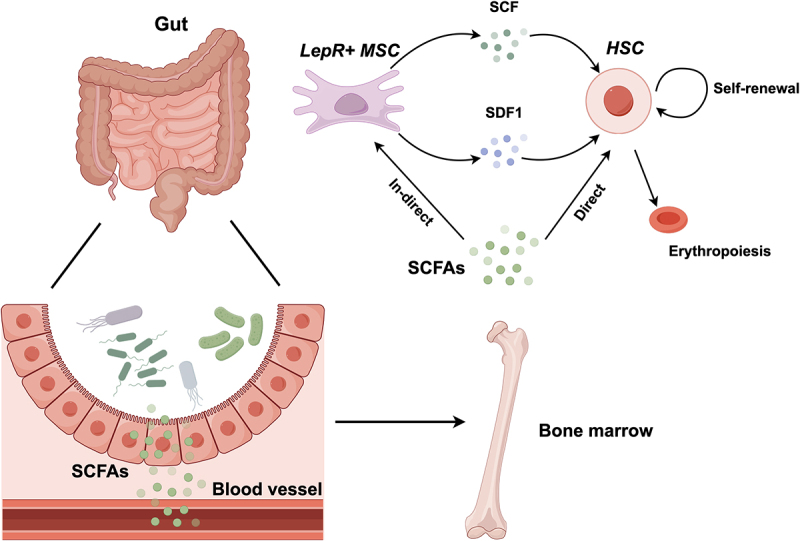
Modulation of HSCs function by SCFAs.

HSCs, hematopoietic stem cells; SCFAs, short-chain fatty acids; LepR^+^ MSC, leptin-receptor-expressing mesenchymal stem cells; SCF, stem cell factor; SDF1, stromal cell derived factor 1.

SCFAs are extensively studied metabolites, primarily generated through the digestion of dietary fiber by the gut microbiota in the host. They play a crucial role in maintaining normal physiological functions of the intestine and the morphology and functionality of colonic epithelial cells.^136^ Additionally, a substantial body of research supports the beneficial effects of SCFAs in regulating host immune function and controlling various diseases, including malignancies.^137–141^ SCFAs, acting as agonists, bind to cell surface G-protein-coupled receptors (GPCRs) to exert their functions. Another role is serving as histone deacetylase inhibitors (HDACIs). Inside the cell, SCFAs inhibit nuclear class I histone deacetylases (HDACs) on one hand and activate histone acetyltransferases (HATs) on the other, thereby influencing cellular genome transcription.^135^ Utilizing SCFAs for the regulation of the host’s hematopoietic system is both an effective and controllable therapeutic approach. The active metabolic byproduct SCFA, butyrate, has the capability to induce the generation of Foxp3^+^ Treg cells.^142^ Butyrate directly influences T cells, thereby enhancing the generation of extrathymic Treg cells. In addition, butyrate plays a role in histone modification, targeting the Foxp3 locus and promoting acetylation of both the locus and the Foxp3 protein. Moreover, as a type of HDACI, butyrate facilitates the induction of Foxp3^+^ Treg cells by suppressing the expression of pro-inflammatory cytokines by DCs. This collaborative action of the gut microbiota helps maintain a balance between pro- and anti-inflammatory cells, fostering mucosal tolerance, mitigating uncontrolled intestinal inflammation, and serving as a preventive measure against inflammatory bowel diseases. Lactate, an end product of gut microbiota metabolism, engages with its cell surface receptor GPCR81. This molecular interaction results in the transcriptional and protein-level downregulation of SCF secretion by LepR^+^ MSCs in the bone marrow, thereby diminishing the self-renewal capacity of HSCs. Concurrently, the activation of GPCR81 on the surface of HSCs by lactate manifests in a discernible attenuation of both self-renewal and differentiation functions^114^ (Figure 3 shows how SCFAs regulate HSC).

Butyrate, as a SCFAs, exerts influence on the kinetics of erythroid lineage growth by eliciting the differentiation of erythroid progenitor cells. At the transcriptional level, it induces an upregulation in the expression of globin genes, particularly γ-globin, during embryonic developmental stages. The oral administration of short-chain fatty acid derivatives (SCFADs) emerges as a viable therapeutic strategy for β-hemoglobinopathies and other forms of anemia. In summary, these observations affirm the favorable impact of SCFAs, released subsequent to the microbial fermentation of dietary fiber in the gut, on the generation of erythroid cells originating from the bone marrow.^143–145^ Mice subjected to a high-fiber diet displayed an augmented spectrum of SCFAs, predominantly characterized by an elevation in butyrate levels. Concurrently, this dietary intervention induced discernible modifications in bone marrow hematopoietic functionality, marked by an amplified production of precursors of monocytes and DCs.^65^ The expanded monocyte population exhibited a distinctive rise in Ly6c^−^ monocytes.^146^ Upon homing to the lungs, these monocyte-derived macrophages demonstrated a reduced capacity to produce the chemokine CXCL1. Given the pivotal role of CXCL1-mediated neutrophil recruitment in the pathogenesis of severe lung tissue damage following influenza virus infection, the selective activation of bone marrow monocytes by dietary fiber stands as a prophylactic strategy against chronic inflammatory disorders in the host.

Bile acids, synthesized by the liver from cholesterol, undergo dynamic circulation within the human body primarily through the enterohepatic circulation system. This metabolic process is subject to the influence of intestinal microbiota. Primary bile acids secreted into the colon can, under the action of intestinal bacteria, catalyze the generation of secondary bile acids (SBAs). For instance, certain genera within the Clostridiales order are capable of mediating the 7α-dehydroxylation of bile acids, leading to the formation of SBAs.^147^ In mice with elevated serum levels of SBAs, the quantity of GMPs in the bone marrow is significantly higher compared to mice with lower levels of SBAs. Studies on bone marrow treated with SBAs reveal an increase in the number of colony-forming units of granulocyte-macrophage (CFU-GM). Further experiments demonstrate that isolated HSPCs treated with SBAs exhibit an enhanced ability to increase CFU-GM, suggesting that direct exposure of HSPCs to SBAs is sufficient to stimulate an increase in GMPs.^148^ Another intriguing aspect is the protective effect of SBAs on HSCs during the fetal period when they are in an expanding state. HSCs cultured in vitro are highly sensitive to endoplasmic reticulum (ER) stress, which is driven by unfolded proteins, leading to apoptosis under prolonged ER stress conditions. The addition of tauroursodeoxycholic acid, a type of bile acid, to the in vitro culture system reduces the severity of ER stress and improves cell survival. During embryonic development, a significant proportion of HSCs settle in the liver and undergo a rapid expansion phase, a process that also benefits from the protective effects of bile acids. In the liver of embryonic mice, a distinctive bile acid metabolism pattern is detected. Their bile acids exhibit a mixture of self-produced primary bile acids and maternal-derived SBAs. Higher bile acid production in this context induces lower ER stress levels, thereby reducing the apoptotic pathways in embryonic liver HSCs.^149^

## 5. Concluding remarks

In summary, the relationship between the gut microbiota and hematopoiesis has transcended mere speculation, emerging as a highly scrutinized and dynamic field of research. Substantial evidence indicates that the gut microbiota intricately interfaces with the hematopoietic system, establishing a close connection through the gut-blood axis. The study of how the microbiota regulates hematopoiesis has evolved from observational and descriptive stages to a more advanced phase involving in-depth mechanistic exploration and the development of intervention strategies. Additionally, attention should be extended to various cellular components integral to the hematopoietic stem cell microenvironment and the foundational interactions among diverse HSCs. The ongoing advancements in this field hold the potential to pioneer novel avenues in the treatment of disorders associated with the hematopoietic system.

## Data Availability

Materials described in the manuscript will be freely available to any scientist wishing to use them for noncommercial purposes, without breaching participant confidentiality.
